# A Menthol-Enhanced “Cooling” Energy Gel Does Not Influence Laboratory Time Trial Performance in Trained Runners

**DOI:** 10.3390/nu15153379

**Published:** 2023-07-29

**Authors:** Roxanne M. Vogel, Nicole Varone, Cayla Clark, Kyndall Ramirez, Megan L. R. Ross, Christian Swann, Christopher J. Stevens

**Affiliations:** 1 Physical Activity, Sport and Exercise Research (PASER) Theme, Faculty of Health, Southern Cross University, Coffs Harbour, NSW 2450, Australia; 2 GU Energy Labs, Berkeley, CA 94710, USA; 3 School of Health Promotion & Kinesiology, Texas Woman’s University, Denton, TX 76201, USAkyndall.ramirez81@gmail.com (K.R.); 4 Australian Catholic University, Melbourne, VIC 3065, Australia

**Keywords:** thermoregulation, supplement, endurance, cooling, cognitive, mint, perception, sports nutrition

## Abstract

l-menthol (menthol) is an organic compound derived from peppermint which imparts a refreshing mint flavor and aroma to oral hygiene products, chewing gum, and topical analgesics. Menthol has been identified as a non-thermal sensory cooling strategy for athletes when ingested or mouth-rinsed during exercise in hot environments. Therefore, sports nutrition products delivering a controlled concentration of menthol could be beneficial for athletes exercising in the heat. We sought to test the performance and perceptual outcomes of a novel menthol energy gel during treadmill running in the heat (33 °C, 49% RH). Fourteen trained runners (mean ± SD; age: 31 ± 6 years, VO_2max_: 56.5 ± 10.1 mL·kg^−1^·min^−1^, BMI: 23.2 ± 2.4 kg/m^2^; six female) participated in a randomized, crossover, double-blind, and placebo-controlled study. A menthol-enhanced energy gel (0.5% concentration; MEN) or flavor-matched placebo (PLA) was ingested 5 min before and again at 20 and 40 min of a 40 min treadmill exercise preload at 60% VO_2max_, followed by a 20 min self-paced time trial. The total distance, vertical distance, perceptual measures (thermal comfort, thermal sensation, rating of perceived exertion, and affect), and cognitive performance via computerized neurocognitive assessment were measured. No difference between 20 min self-paced time trial total distance (MEN: 4.22 ± 0.54 km, PLA: 4.22 ± 0.55 km, *p* = 0.867), vertical distance (MEN: 49.2 ± 24.6 m, PLA: 44.4 ± 11.4 m, *p* = 0.516), or any perceptual measures was observed (all *p* > 0.05). Cognitive performance was not different between the trials (all *p* > 0.05). These results suggest that a menthol energy gel is not superior to a non-menthol gel in terms of performance or perception during treadmill running in the heat. More research is needed to confirm whether these findings translate to ecologically valid settings, including outdoor exercise in ambient heat and during competition.

## 1. Introduction

Performance decrements that occur during prolonged exercise in the heat have been well documented [1,2,3,4]. Thermal stress imposes an additional challenge to the cardiovascular system’s ability to maintain a normal core temperature (37 ± 0.5 °C) [5,6,7]. As core temperature rises, eventually athletes are forced to reduce their power output or cease activity altogether due to heat-induced fatigue or symptoms of hyperthermia [4,8]. Important to the process of thermoregulation during exercise is thermal perception, commonly measured using psychometric scale ratings of thermal sensation and thermal comfort [9]. During prolonged exercise in the heat, warm and uncomfortable perceptions develop, which ultimately impact performance and perceived exertion [10]. Altering thermal perception can modify behavioral thermoregulation, such that self-selected work output and pacing are positively influenced [11]. Cooling methods for athletes have traditionally aimed to reduce the physical body temperature, such as ice vests and collars, ice slurry ingestion, and cold water immersion, while improving thermal perception has been a beneficial secondary outcome [12]. However, the use of non-thermal sensory cooling methods can improve thermal perceptions without any change in the thermal state (i.e., reduction in physical body temperature) [9]. It remains to be seen whether changes in thermal perceptions have a causative effect on performance.

A non-thermal cooling method that has gained attention in recent years involves the internal application of l-menthol (menthol). Mouth rinsing or ingestion of menthol can positively impact thermal comfort and thermal sensation during endurance exercise performed under hot conditions [13]. In both cycling [14,15,16,17] and running [18,19], mouth rinsing with 25 mL of 0.01–0.1% menthol solutions has shown promise in improving the time to exhaustion (6–9% increase [15,16,17]) and time trial performance (2.7–3.8% improvement [18,19]) in the heat [13]. Menthol ingestion (25 mL every 5 min, 0.01% concentration) also improved breathing comfort and exercise capacity during treadmill running to exhaustion under thermoneutral conditions (20.2 °C, 66% RH) [20]. Additionally, a single menthol mouth rinse (25 mL, 0.1% concentration) improved relative the power output during high intensity, short duration (3 min) cycling in the heat (33 °C, 46% RH) compared to both placebo and cold water mouth rinsing [21]. Hence, menthol may be ergogenic in a variety of sports nutrition applications.

Despite a growing body of research on the ergogenic potential of menthol mouth rinsing and ingestion, commercially available menthol products are not widely distributed and have a low concentration of menthol (i.e., <0.1%), which may not maximize menthol’s perceptual cooling benefits and might even encourage athletes to improvise their own, unsafe products [22]. This is of concern to athletes who require efficacious, third-party tested supplements that can be used during sanctioned competition and are considered safe [22]. Furthermore, the ideal menthol concentration, dosage, and application protocol remain to be elucidated.

Previously, we have developed, optimized, and demonstrated that a menthol-enhanced energy gel at a 0.5% concentration imparts a perceptual cooling sensation lasting up to 20 min during exercise in warm/humid outdoor conditions [23,24]. The optimized gel, which produced a significantly greater cooling sensation than lower-concentration menthol gels (e.g., 0.1% and 0.3%) without causing irritation, was an improvement on a previous version which had been deemed “too strong” in flavor and therefore unacceptable to athletes [23]. The purpose of the present study was to determine whether the optimized menthol energy gel could influence performance and perceptions during running in the heat. A secondary aim was to assess the effects of a menthol gel on cognitive performance following exercise.

## 2. Materials and Methods

Sixteen non-heat-acclimated, trained male and female runners were recruited for this randomized, double-blind, crossover, and placebo-controlled study. An a priori power calculation (G*power, v. 3.1.9.4) indicated 12 participants would be required for a one-way ANOVA analysis with the alpha set at 0.05, power of 0.80, and an effect size of 0.4 for the time trial performance distance covered (km), as derived from a previous study evaluating the effects of menthol mouth rinsing on 3 km running performance [19]. Ethical approval was granted by Southern Cross University (approval #2021/125) and Texas Woman’s University (approval #FY2022-103), and the research was conducted in accordance with the Declaration of Helsinki. Participants were recruited via word-of-mouth advertising on campus and at local running groups in the Denton, Texas area.

The inclusion criteria for participants were the following: (a) 18–40 years of age, (b) healthy, as assessed by the Exercise and Sport Science Australia adult pre-exercise screening tool [25], (c) endurance trained (i.e., consistently performing vigorous endurance exercise training sessions >30 min, at least three days/week over the previous three months), (d) English speaking, and (e) experienced running on a treadmill. The exclusion criteria included: (a) presence or history of a medical condition that may have impacted participant safety during the study, including cardiovascular, metabolic, renal, or hepatic disease, and/or musculoskeletal injury, (b) presence of anosmia or dysgeusia, (c) use of any medicine or supplements that could significantly affect the study outcome (e.g., diuretics, antihistamines, amphetamines, thyroid medication), (d) known allergy or intolerance to any of the ingredients contained in the energy gel, (e) a history of exertional heat illness, (f) currently pregnant or planning to become pregnant during the course of the study, and/or (g) had visited a hot climate for longer than one week within the two months prior to participation. All the experimental visits were conducted in January–April 2022 to coincide with the cooler ambient months in the Northern Hemisphere.

Participants visited the laboratory on three occasions: a preliminary testing and familiarization visit, experimental visit 1, and experimental visit 2. Visits were scheduled at least 72 h apart and at the same time of day (±1 h) to avoid any influence of diurnal variation. Participants were instructed to refrain from exercise and the consumption of alcohol or ergogenic aids for 24 h, and from engaging in behaviors (e.g., sauna bathing) that might otherwise influence body temperature or induce fatigue. The use of mints, chewing gum, mouthwash, or any peppermint-containing products was prohibited within 2 h of each visit. Furthermore, participants were instructed to refrain from strenuous exercise for 48 h prior to each visit and to replicate their sleep regimen the night before each trial. Participants filled out a 24 h dietary recall log before each experimental visit and were instructed to repeat the same dietary intake 24 h before each subsequent visit. Dietary recall logs were compared before the experimental trials to ensure similar dietary intake prior to data collection.

### 2.1. Baseline Testing and Familiarization

During the baseline testing and familiarization visit, participants completed a trial of a neurocognitive assessment (DANA Brain Vital, AnthroTronix, Silver Spring, MD, USA) and an abbreviated Profile of Mood States questionnaire [26] on a handheld tablet, as detailed below. Participants were briefed on the maximal aerobic capacity (VO_2max_) test protocol and familiarized with the Borg CR-10 rating of perceived exertion (RPE) scale [27]. The VO_2max_ was determined via a graded incremental treadmill exercise protocol using breath-by-breath analysis on a calibrated metabolic cart (K5, COSMED, Concord, CA, USA). A modified Åstrand incremental exercise protocol was used to determine the VO_2max_, starting with a 5 min warm up stage at a 1.0% incline (TM55, Cardiac Science, Bothell, WA, USA). The treadmill belt speed was gradually increased until reaching the participant’s self-estimated 10 km pace by the end of the stage [28]. Following the warm up, the athletes completed a single 3 min stage at 10 km pace and a 1.0% incline before the incline was increased by 2% every minute until failure, volitional termination, or attaining two or more criteria for the VO_2max_ [29]. A heart rate value corresponding to 60% VO_2max_ was used as the target heart rate during steady state treadmill running for subsequent experimental visits. Baseline tests were conducted under thermoneutral conditions (20 °C, 39% RH).

### 2.2. Experimental Trials

Exercise during the experimental trials took place in an environmental heat chamber (33 °C, 49% RH). Prior to the experimental sessions (4–6 h), participants ingested a core temperature sensor (CorTemp, Palmetto, FL, USA) with water. Participants were encouraged to maintain adequate hydration prior to all testing visits and instructed to consume 500 mL of plain water in the two hours before arriving at the laboratory. Female participants were scheduled to avoid variation in their menstrual cycle, with the second experimental trial scheduled within three days of the first and both scheduled to coincide with the follicular phase, as based on self-reported cycle tracking. Urine specific gravity (USG) was assessed from a urine sample provided upon arrival at the laboratory for all visits via a handheld refractometer (Atago, Bellevue, WA, USA). Water was provided to participants if the urinalysis results indicated mild to moderate dehydration (i.e., USG > 1.020) and USG was re-evaluated before commencing any procedures. Body mass was measured in lightweight running clothing (e.g., shorts, singlet, socks) before participants were fitted with a heart rate monitor (HRM-Run, Garmin, Olathe, KS, USA) and asked to remain seated quietly for 10 min prior to baseline assessments of their heart rate, blood pressure, and core temperature (T_core_). Baseline assessments of cognitive function and the Profile of Mood States questionnaire were completed immediately after the physiological assessments. After all the baseline measures were completed, participants ingested the assigned energy gel (PLA or MEN), “swishing” the contents of the gel around their mouth for 5 s before swallowing, followed by 150 mL of tepid (20–22 °C) water. Details of the gel’s composition are provided below. Subsequent gels were ingested using the same protocol.

Participants completed 40 min of steady state running in the heat (33 °C, 49% RH) on a motorized treadmill (505 CST, ProForm, Logan, UT, USA) at a heart rate corresponding to 60% of the maximal value observed at VO_2max_. To maintain the desired intensity over the 40 min preload, the research team monitored the participants’ heart rate and adjusted the treadmill speed accordingly until a steady state was achieved. Tepid (20–22 °C) water was offered every 10 min, and a fan continuously circulated the chamber’s air at a speed of 2 m·s^−^^1^.

The gels were administered with 150 mL of tepid water at 20 min and following 40 min of the steady-state preload. The thermal sensation (TS), thermal comfort (TC), RPE, affect, T_core_, and heart rate (HR) were measured and recorded every 10 min throughout the protocol. After the 40 min preload, participants completed the neurocognitive tests and mood questionnaires on a handheld tablet while seated in the heat chamber prior to ingesting the third gel with 150 mL of water. Following this (~5 min after finishing the exercise preload), participants completed a 20 min, self-paced time trial (TT). Participants were instructed to cover as much distance as possible and were able to freely control the speed of the treadmill, although they were blinded to the speed, pace, and overall distance covered. The maximal speed of the treadmill was 16.1 km/h, and participants were instructed to only increase the incline if the treadmill’s maximal speed was not sufficient. The treadmill was otherwise set at a 1.0% incline throughout all the trials. The total distance (km) and vertical distance (m) were recorded from the TM at the end of the 20 min TT. Measurements of the TC, TS, RPE, affect, T_core_, and HR were recorded at 10 min and at the end of the TT. Participants completed the cognitive/mood assessments immediately upon completing the TT while seated inside the heat chamber. Tepid water was available ad libitum during this time. Upon completing the assessments, participants exited the environmental chamber and their body mass was recorded after towel drying any sweat from the body. Participants remained seated in a thermoneutral room for at least 15 min to monitor their recovery status, during which their heart HR, T_core_, and blood pressure were assessed.

#### 2.2.1. Performance, Perceptual, and Cognitive Measure

Performance was measured as the total (km) and vertical (m) distance recorded during the 20 min TT. Thermal sensation (TS) was rated on a 1–13 visual analogue scale anchored by the words “unbearably cold” at 1 and “unbearably hot” at 13, and with “neutral” associated with a value of 7 [30]. Participants were prompted by the question: “How does the temperature of your body feel?” Thermal comfort (TC) was rated on a 1–10 scale, with 1 indicating “comfortable” and 10 indicating “extremely uncomfortable”, prompted by the question: “How comfortable do you feel with the temperature of your body?” [31]. The total mood disturbance (TMD) was derived from the abbreviated Profile of Mood States (POMS) scale score of 40 adjectives that measure tension, depression, fatigue, vigor, confusion, anger, and esteem-related affect. Words such as “energetic”, “grouchy”, and “weary” were rated on a 5 pt scale from zero indicating “Not at all” to 4 indicating “Extremely” [26]. RPE was rated on a 1–10 scale, with 10 indicating maximal exertion [27]. Affect was rated on the Feeling Scale from −5 (very bad) to +5 (very good), with zero indicating neutral, and prompted by the question “How do you feel overall right now?” [32].

Neurocognitive performance (reaction time, percent correct, and cognitive efficiency) was assessed using DANA Brain Vital through the following three tests:

1. Simple Reaction Time: The participant tapped on a non-moving, circular target as quickly as possible each time it appeared on the screen over 40 trials.
2. Procedural Reaction Time: The screen displayed one of four numbers (2, 3, 4, or 5) for two seconds. The participant tapped the left button (labelled “2 or 3”) or right button (labelled “4 or 5”) at the bottom of the screen as quickly as possible to indicate which number was displayed over 32 trials.
3. Go–No Go: A house was presented on the screen with several windows. Either a “friend” (green character) or “foe” (grey character) appeared in a window. The participant tapped the screen only when a “foe” appeared over 30 trials.

#### 2.2.2. Energy Gels

Energy gels with a 0.5% menthol concentration, as determined from and described in a previous investigation [24], were produced by a commercial sports nutrition manufacturer (GU Energy Labs, Berkeley, CA, USA) using the following ingredients: maltodextrin, water, fructose, L-leucine, sodium citrate, medium-chain triglycerides, sea salt, potassium citrate, citric acid, calcium carbonate, L-valine, gellan gum, L-isoleucine, sodium benzoate (preservative), potassium sorbate (preservative), natural L-menthol (Sigma-Aldrich, St. Louis, MO, USA), and natural citrus flavor. A placebo gel was also produced containing a natural mint flavor in place of the l-menthol (Virginia Dare, Brooklyn, NY, USA), as previously described [23]. All the gels contained 11 g of total carbohydrate, were non-caffeinated, and were provided in 16 g single-use packets. A member of the supplement manufacturer’s research and development team not involved with data collection assigned codes to each gel to conceal the identity from the investigators and participants until the data analysis was complete.

### 2.3. Statistical Analysis

All analyses were conducted using SPSS Statistics (version 28.0; IBM Corp., Armonk, NY, USA). A one-way ANOVA was used to analyze variables measured at a single time point (i.e., total distance, vertical distance). Data recorded pre, mid, post, and/or at 10 min intervals were analyzed via a two-way, repeated-measures ANOVA (time × treatment), with pairwise comparisons performed using a Bonferroni correction. Normality was assessed by Shapiro–Wilk tests and sphericity was assessed using Mauchly’s test of sphericity. When sphericity could not be assumed, a Greenhouse–Geisser correction was applied. Cohen’s d was calculated for primary outcome variables and interpreted as trivial, small, moderate, and large for effect sizes between 0.0–0.19, 0.2–0.6 SD, 0.6–1.2 SD and >1.2 SD, respectively. [33]. Statistical significance was set at *p* < 0.05, and data are presented as the mean ± standard deviation.

## 3. Results

Participants’ demographic information is presented in Table 1. Sixteen runners (eight female) volunteered to participate in the study. One participant discontinued due to an injury sustained unrelated to the study, while another was excluded from the final analysis after informing the investigators she had not performed at maximal effort in the TT. Therefore, 14 participants (six female) were included in the data analysis. Environmental chamber conditions during the experimental visits were not significantly different between trials (MEN: 33.2 ± 0.7 °C, 49.1 ± 2.3% RH vs. PLA 33.0 ± 0.7 °C, 48.6 ± 2.8 % RH; *p* > 0.05). During the 40 min preload, the treadmill speed (MEN: 5.92 ± 1.03 km/h vs. PLA 5.76 ± 0.97 km/h; *p* = 0.76) and cumulative distance covered (MEN: 3.95 ± 0.66 km vs. PLA 3.87 ± 0.63 km; *p* = 0.99), as recorded at 10 min intervals, were not significantly different. During the 20 min TT, two participants reached the maximal treadmill speed and subsequently increased the incline, although neither one maintained the additional incline for the duration of the trial once they added it, eventually decreasing it back to 1%.

**Figure 1:**
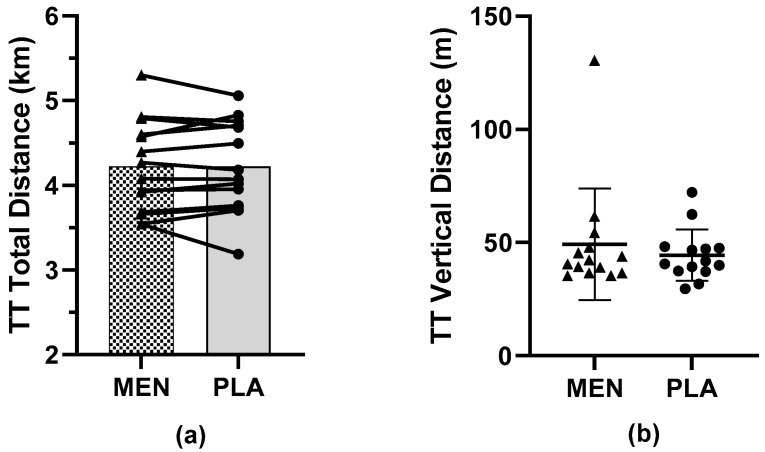
Individual results for (**a**) total distance and (**b**) vertical distance covered during 20 min time trial (TT). Results presented as individual responses (triangles/circles) and mean ± SD. MEN = menthol, PLA = placebo.

Time trial performance is illustrated in Figure 1. There was no significant difference between MEN (4.22 ± 0.54 km) and PLA (4.22 ± 0.55 km) for the total distance (*p* = 0.867), nor for the total vertical distance covered during the TT (*p* = 0.556). The effect size was trivial for the total distance (d = 0.00) and small for the vertical distance (d = 0.25). There was no trial order effect when the TT performance was assessed as experimental visit 1 (4.22 ± 0.58 km) compared to experimental visit 2 (4.22 ± 0.51 km, *p* = 0.99), regardless of the intervention order.

**Figure 2:**
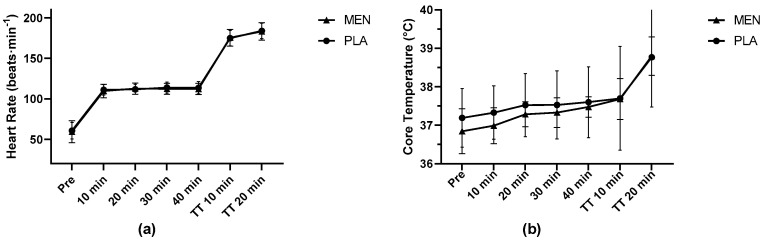
Responses for (**a**) heart rate and (**b**) core temperature during treadmill exercise protocol. Results presented as mean ± SD. TT = time trial, MEN = menthol, PLA = placebo.

The responses for the T_core_ and HR are illustrated in Figure 2. There were no significant differences between the conditions for any physiological measure. There was a main effect for time for the HR, with both the MEN and PLA exhibiting an increase over time (*p* < 0.001), but no significant interaction of treatment by time (*p* = 0.539). Similarly, the T_core_ increased over time in both the MEN and PLA conditions (*p* < 0.001), with no significant interaction of treatment by time (*p* = 0.421).

**Figure 3:**
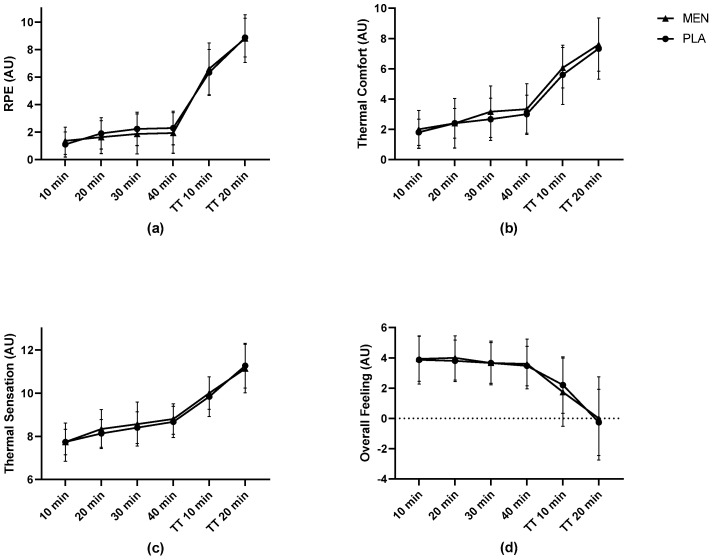
Participant ratings of (**a**) RPE, (**b**) thermal comfort, (**c**) thermal sensation, and (**d**) affect during treadmill exercise protocol. Results presented as mean ± SD. AU = arbitrary units, TT = time trial, MEN = menthol, PLA = placebo.

The perceptual responses are illustrated in Figure 3. There were no significant differences between the conditions for any perceptual measure. The RPE increased significantly over time in both the MEN and PLA (*p* < 0.001), with no difference between the groups over time (*p* = 0.584). The TC similarly increased over time in both conditions (*p* < 0.001), with no significant effect of group by time interaction (*p* = 0.920). The TS increased over time in the MEN and PLA trials (*p* < 0.001), with no difference between the groups over time (*p* = 0.733). Finally, the affect scores decreased similarly in both conditions over time (*p* = 0.016), with no significant interaction of treatment over time (*p* = 0.346).

**Figure 4:**
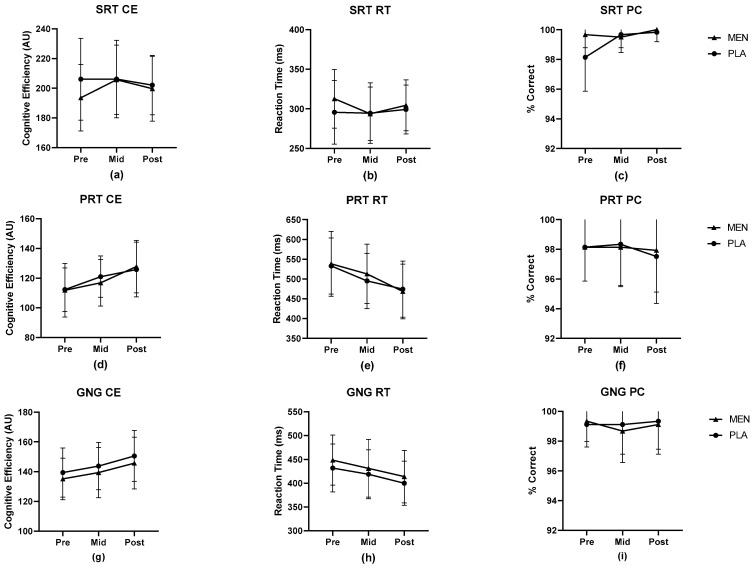
Cognitive performance results for computerized assessments of the Simple Reaction Time task (**a**–**c**), Procedural Reaction Time task (**d**–**f**), and Go–No Go task (**g**–**i**) taken before (pre), during (mid), and after (post) the treadmill exercise protocol. Results presented as mean ± SD. CE = cognitive efficiency, RT = reaction time, PC = percent correct, AU = arbitrary units, MEN = menthol, PLA = placebo.

The cognitive performance data are illustrated in Figure 4. There were no significant differences between the conditions for any cognitive performance measure. Simple Reaction Time results for the reaction time (RT), percent correct (PC) and cognitive efficiency (CE) revealed a main effect for time but no significant interaction effect (all *p* > 0.05). The Procedural Reaction Time and Go–No Go test results similarly revealed a main effect for time but no significant interaction effect for RT, PC, or CE (all *p* > 0.05).

**Figure 5:**
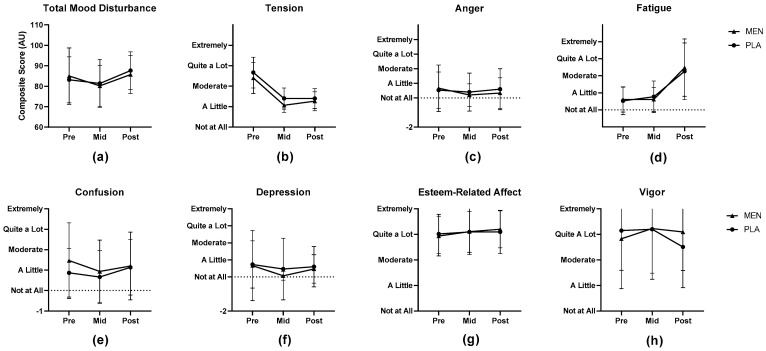
Profile of Mood States (POMS) assessment results for (**a**) total mood disturbance and POMS subscales of (**b**) tension, and (**c**) anger, (**d**), fatigue, (**e**) confusion, (**f**) depression, (**g**) esteem-related affect, and (**h**) vigor taken before (pre), during (mid), and after (post) the exercise protocol. Results presented as mean ± SD. AU = arbitrary units, MEN = menthol, PLA = placebo.

The Profile of Mood States data are illustrated in Figure 5. There was a main effect for time for the TMD (*p* = 0.018) but no significant interaction effect (*p* > 0.05). Tension (*p* < 0.01) and fatigue (*p* < 0.001) similarly displayed main effects for time but not for the interaction of group by time (*p* > 0.05). The remaining POMS subscales (i.e., anger, depression, esteem-related affect, vigor, and confusion) did not exhibit significant effects for time, treatment, or the interaction of treatment by time (all *p* > 0.05).

## 4. Discussion

This is the first study of its kind to examine the performance and perceptual effects of a menthol-enhanced energy gel during exercise in the heat. The main finding was that the menthol energy gel was not superior to a placebo gel in terms of the 20 min time trial running performance, physiology, perceptions, or cognitive performance in trained runners following 40 min of moderate treadmill running under heat stress. While the results are largely in contrast to previous research on menthol use in sport, the current findings may be limited to indoor exercise scenarios, the protocol employed, and the specific characteristics of the participants (i.e., trained, non-heat-acclimated, recreational runners).

The potential benefits of menthol use during endurance exercise in the heat include improved thermal comfort and sensation, reduced RPE, and enhanced time trial/time to exhaustion performance [13,34], although the results of the current study did not demonstrate an ergogenic effect of MEN compared to PLA. In our study, the menthol delivery format (energy gel) was substantially different compared to previous studies, which have involved menthol mouth rinsing (without ingestion) or menthol-flavored beverages. However, our findings align with a recent study which found no difference between menthol mouth rinsing and placebo on the 10 km time trial performance in recreationally trained runners [35]. In that study, performed indoors under thermoneutral conditions (22.5 °C, 39.2% RH), repeated application of a 0.01% menthol mouth rinse and 8% menthol topical cream or a placebo treatment produced similar benefits versus the control, improving 10 km treadmill run time by ~2.5% (1.16 min) for the menthol and ~2.8% (1.3 min) for the placebo [35]. These findings suggest a potential role for the ergogenic effects of taste and a possible interplay with the placebo effect, a phenomenon which has been highlighted in recent work by Best and colleagues [36]. Although our study did not have a control arm, it is also possible that participants experienced an ergogenic effect of taste and/or a placebo effect, since both the MEN and PLA were similarly flavored.

Unlike previous studies conducted among endurance athletes [17,18,19], the menthol energy gels in the current study also contained carbohydrate (CHO), a known ergogenic aid, which may have overshadowed the potential benefits of menthol cooling. It has been established that CHO sensing in the oral cavity can enhance performance, largely through stimulating neural reward systems that impact motivation and lower perceived effort [37]. Since the perceptual benefits of menthol cooling rely similarly on the activation of oral receptors and centrally mediated mechanisms, it may be that the performance effects of the two (CHO + menthol) are not additive. In a study comparing menthol, CHO, and combined (menthol + CHO) mouth rinsing, there was no additive benefit to the combined swill during a 40 km cycling time trial in the heat [38]. Similarly, a menthol-enhanced sports drink produced no difference in the time to exhaustion after 60 min of intense indoor cycling compared to a standard CHO beverage under thermoneutral conditions [39]. The authors speculated that the product’s low menthol concentration (0.01%) was insufficient to produce a substantial cooling effect, thus limiting any ergogenic potential. In considering the studies with both CHO and menthol provision, it may be that during intense endurance exercise, glycogen depletion and the brain’s perception of resource availability (i.e., CHO provision) are of primary concern, thus taking priority over the effects of menthol’s sensory cooling properties.

There was no significant perceptual benefit for the MEN vs. PLA in terms of TS, TC, RPE, or affect. The perceptual cooling effect of menthol during use in sport has been observed at lower concentrations (i.e., 0.01–0.1% menthol) by previous investigators [16,17,18,19]. The menthol gels in the current study, by contrast, contained a higher concentration (0.5% menthol) and have been demonstrated to impart a cooling sensation lasting up to 20 min when ingested prior to running in warm outdoor conditions (27.5 ± 6.8 °C, 58.2 ± 23.2% RH) [24]. The environmental conditions in the current study were hotter (i.e., 33 °C, 49% RH), and it is possible that the thermal stress that was produced (see Figure 2b) was too high, thereby outweighing any menthol sensory cooling that would notably impact perceptual measures. The protocol employed in the present study was selected to elevate the T_core_ prior to an intense, short-duration effort similar to that experienced during a 5 km footrace. Furthermore, participants reported feeling increasingly hot and less comfortable over time during the protocol, as is reflected in their TC and TS ratings (Figure 3b and Figure 3c, respectively). While our protocol did appear to produce high thermal stress, a reliable 60 min treadmill protocol for inducing significant thermal stress has recently been reported [40], and future investigations may seek to compare a menthol-enhanced gel versus a standard energy gel with this protocol.

Finally, there was no difference in the POMS scores or cognitive performance in terms of the reaction time, accuracy, or cognitive efficiency or when comparing the MEN and PLA during and after exercise. These results agree with previous investigations of the perceptual and cognitive effects of menthol during exercise in the heat. Saldaris and colleagues [41] found no benefit to cognitive performance or mood with menthol mouth rinsing (25 mL, 0.1% concentration) every 15 min during prolonged treadmill running in the heat (35 °C, 59% RH). In another study, no improvement in alertness or cognition was observed following the ingestion of 30 mg of menthol lozenges during a simulated firefighting task performed in the heat (35 °C, 40% RH) [42]. Menthol and peppermint extract have been suggested to positively influence central nervous system function in a manner pertinent to sport, potentially by modulating dopaminergic function and/or the positive allosteric modulation of GABA_A_ receptors [43]. However, results supporting this notion have thus far been limited to non-exercise scenarios [44,45] and rodent models [46,47].

The strengths of the current study include a well-controlled laboratory setting and randomized, double-blind, and placebo-controlled study design, although it was not without limitations. The lack of familiarization with the preloaded time trial protocol in the heat chamber may have introduced a learning effect across the two experimental visits, although the subsequent analysis demonstrated that there was no trial order effect on time trial performance between the first and second visits. The maximum speed of the treadmill could also be considered a limitation, which was corrected for by allowing participants to increase the gradient of the incline upon reaching the maximal speed. Therefore, the total vertical distance covered was also measured as a performance outcome. Female participants were scheduled to avoid variation in their menstrual cycle, with the second experimental trial scheduled within three days of the first, and both were scheduled to coincide with the follicular phase, when the T_core_ is lower [48]. This was based on self-reported cycle tracking and not a direct measurement of the hormonal milieu, making it possible that some of the female participants undertook the second experimental trial in a different phase (e.g., early luteal vs. late follicular), which can impact the T_core_, thermoregulation, and thermal sensation [47]. However, the fluctuation in the T_core_ over the course of the menstrual cycle has not been found to consistently influence aerobic performance [49].

Despite recruiting only trained runners from campus and local area running clubs, the maximal aerobic capacity (VO_2max_) of the participants ranged from 41.6 to 75.1 mL·kg^−^^1^·min^−^^1^. Although all the participants were currently running three or more days per week, there was a wide range of experience within the sample group, ranging from competitive (Tier 3) to recreational (Tier 1) runners, as classified by McKay et al. [50]. It is known that more highly trained athletes are better able to tolerate heat than lesser trained individuals [51], a factor which may have impacted the perceptual responses and performance more dramatically for some participants than others. Last, although every effort was made to blind the researchers and participants to the identity of the gels, it is possible that participants were able to guess which gel contained menthol. Following the experimental visits, 11 out of 14 participants correctly guessed the identity of the menthol gel.

## 5. Conclusions

Among recreationally trained runners, a menthol-enhanced energy gel did not provide superior performance or perceptual benefits during a 20 min time trial following 40 min moderate treadmill exercise in the heat compared to a non-menthol energy gel. More research is needed to confirm whether these findings are consistent in ecologically valid scenarios, such as outdoor competition in the heat, and among elite athletes.

## Figures and Tables

**Table 1:** Participant demographics.

	All (*n* = 14)	Male (*n* = 8)	Female (*n* = 6)
Age (years)	30.9 ± 5.7	31.5 ± 6.2	30.2 ± 5.4
Height (cm)	172.27 ± 7.99	176.81 ± 5.57	166.22 ± 6.74
Weight (kg)	69.21 ± 11.64	73.98 ± 12.32	62.87 ± 7.42
BMI (kg·m^−2^)	23.18 ± 2.36	23.58 ± 2.95	22.68 ± 1.4
VO_2max_ (L·min^−1^)	3.88 ± 0.82	4.3 ± 0.84	3.31 ± 0.31
VO_2max_ (mL·kg^−1^·min^−1^)	56.52 ± 10.1	59.21 ± 12.18	52.93 ± 5.5

## Data Availability

Supporting data available upon request.
